# Development and validation of a new Arrhythmia-Specific questionnaire in Tachycardia and Arrhythmia (ASTA) with focus on symptom burden

**DOI:** 10.1186/1477-7525-10-44

**Published:** 2012-04-30

**Authors:** Ulla Walfridsson, Kristofer Arestedt, Anna Stromberg

**Affiliations:** 1 Division of Nursing Science, Department of Medicine and Health Sciences, Linkping University, Department of Cardiology UHL, County Council of stergtland, Linkping, Sweden; 2 School of Health and Caring Sciences, Faculty of Health, Social Work and Behavioural Sciences, Linnaeus University Kalmar and Department of Medical and Health Sciences, Division of Nursing Science, Linkping University, Linkoping, Sweden; 3 Division of Nursing Science, Department of Medicine and Health Sciences, Linkping University, Department of Cardiology UHL, County Council of stergtland, Linkping, Sweden; 4 Department of Cardiology, University Hospital, SE 581 85, Linkping, Sweden

**Keywords:** Arrhythmias, Symptom burden, Disease-specific questionnaire, Questionnaire development, Validation

## Abstract

**Background:**

Arrhythmias can appear with a variety of symptoms, all from vague to pronounced and handicapping symptoms. Therefore, patient-reported outcomes (PROs) concerning symptom burden are important to assess and take into consideration in the care and treatment of patients with arrhythmias. The main purpose was to develop and validate a disease-specific questionnaire evaluating symptom burden in patients with different forms of arrhythmias.

**Methods:**

A literature review was conducted and arrhythmia patients were interviewed. Identified symptoms were evaluated by an expert panel consisting of cardiologists and nurses working daily with arrhythmia patients. SF-36 and Symptoms Checklist (SCL) were used in the validation of the new questionnaire Arrhythmia-Specific questionnaire in Tachycardia and Arrhythmia (ASTA).

Homogeneity was evaluated with Spearman´s correlations and Cronbach´s alpha coefficient (α) was used to evaluate internal consistency. Construct validity was evaluated using item-total correlations and convergent and discriminant validity. For this, Spearman´s correlations were calculated between the ASTA symptom scale, SCL and SF-36. Concurrent validity was validated by Spearman´s correlations between the ASTA symptom scale and SCL.

**Results:**

The correlations between the different items in the ASTA symptom scale showed generally sufficient homogeneity. Cronbach´s coefficient was found to be satisfactory (α = 0.80; lower bound 95 % CI for α = 0.76). Construct validity was supported by item-total correlations where all items in the symptom scale were sufficiently correlated (≥0.3). Convergent and discriminant validity was supported by the higher correlations to the arrhythmia-specific SCL compared to the generic SF-36. Concurrent validity was evaluated and there were sufficiently, but not extremely strong correlations found between the ASTA symptom scale and SCL.

**Conclusions:**

The nine items of the ASTA symptom scale were found to have good psychometric properties in patients with different forms of arrhythmias. Arrhythmia patients suffer from both frequent and disabling symptoms. The validated ASTA questionnaire can be an important contribution to PROs regarding symptom burden in arrhythmia patients.

## Background

Heart rhythm disturbances include several forms of arrhythmias. The most common form is atrial fibrillation (AF), occurring in 1–2 % of the general population and with an increasing prevalence in older patients [1]–[4]. Arrhythmias can appear in recurrent attacks or be incessant, are known to be unpredictable and can appear with a variety of symptoms, all from vague to pronounced and handicapping symptoms [4]–[8].

Descriptions of arrhythmia patients symptom burden often include the disease-specific symptoms, frequency, duration and severity where particularly frequent attacks have shown to negatively affect the patients life situation [5]–[8].

Main goals when treating arrhythmia patients can be either curative treatment or to achieve symptom relief. When evaluating disease severity and intervention efficacy it is of great importance to examine not only objective parameters such as ECG-measurements, where also patient-reported outcomes (PROs) are important to take into consideration to assess patients subjective well-being and daily life situation [9]–[12]. Most of the scales or questionnaires available for assessing PROs in arrhythmia patients are designed for AF patients separately, or developed for patients with other supraventricular arrhythmias [13]–[21]. The Symptom Checklist, Frequency and Severity scale (SCL) and the Patient Perception of Arrhythmia Questionnaire (PPAQ) are the only scales that have been used in a wide range of arrhythmia patients. SCL is mostly used in AF patients and PPAQ is designed for other arrhythmias [15,17,21].

Some of the existing questionnaires can be criticized for mixing symptoms and effects of symptoms in the same scale, not including frequency and duration of the episodes and for not examining the presence of the more aggravating symptoms near syncope and syncope.

To the best of our knowledge there is no validated symptom burden questionnaire developed for assessing patient-reported outcomes (PROs) in patients with most different forms of arrhythmias. For this purpose there was a lack of a validated disease-specific symptom questionnaire suitable for most forms of arrhythmias.

### Aims

The aims of the study were to develop and validate a disease-specific symptom questionnaire including a symptom scale for patients with different forms of arrhythmias and to describe patients arrhythmia symptoms.

## Methods

The present study is a part of a project aiming to develop and validate a disease-specific questionnaire suitable for different forms of arrhythmias, the Arrhythmia-Specific questionnaire in Tachycardia and Arrhythmias (ASTA). The questionnaire as a whole is developed to evaluate both symptom burden and the impact of arrhythmia on a patients’ Health-Related Quality of Life (HRQOL). In this article the development and validation of the symptom part, including a symptom scale are described.The Regional Ethical Review Board at the Faculty of Health Sciences, Linköping, Sweden, approved the protocol and gave permission for obtaining a verbal informed consent from the participants. Study participation was documented in the patients’ medical record. The study complies with the Declaration of Helsinki [22,23]. (Number: M170-08 T111-08).

### The development phase

To identify representative symptoms covering different forms of arrhythmia, a comprehensive literature review was conducted in Medline.

In addition, approximately 300 patients with different forms of arrhythmias (most with supraventricular but also some with ventricular arrhythmias) referred for radiofrequency ablation (RFA) were interviewed about their symptoms in connection with arrhythmia episodes. The interviews were conducted before the ablation procedure using a structured questionnaire guide.

From the results items were created by the first author in collaboration with an electrophysiologist.

The ASTA questionnaire was distributed to 6–8 cardiologists, 8–10 nurses at each time of evaluation and over 100 patients were approached by the first author during the development phase, in addition to the interviews.

The symptoms were approved with two changes, one was a complementary explanation recommended for the symptom cold sweat to avoid misinterpretation. The symptom pressure in the chest was extended to be combined with “discomfort”. Based on the identified symptom, a symptom burden scale was created.

The selected symptoms then were: breathlessness during activity, breathlessness even at rest, dizziness, cold sweat, pronounced tiredness, chest pain, pressure/discomfort in chest and worry/anxiety. Three symptoms remained outside the scale; tiredness after having arrhythmia, experiences of near syncope and syncope in connection with the arrhythmia episode.

Symptoms of palpitation, duration and frequency of the arrhythmia attacks were questioned for, also outside the scale.

In the initial phase, the ASTA symptom questionnaire was used in over 500 patients with different forms of arrhythmias: AF and atrial flutter (AFL) patients scheduled for DC-conversion, AF patients randomized to treatment with anti-arrhythmic drugs in comparison to RFA [24] and patients treated with RFA due to AV-Nodal reentry tachycardia (AVNRT), Wolff-Parkinson-White syndrome (WPW), focal atrial tachycardia (FAT), atrial macro-reentry, AFL, AF patients treated with left atrial or His bundle catheter ablation, as well as patients with ventricular arrhythmias (PVCs or VT). The patients were approached when they came for their scheduled intervention, for example DC-conversion, many due to RFA, or to out-patient clinics for scheduled study visits.

Patients were encouraged to comment and suggest additional symptoms if needed. This process resulted in two more symptoms being added to the scale, weakness and infirmity. The symptom worry/anxiety was reworded to “worry” and the vocabulary “tachycardia attacks” was changed to “heart rhythm disturbance” to be suitable for patients with attacks, frequent extra beats or with persistent arrhythmias. The extended 10 item scale was again reviewed and confirmed by the expert panel. During the initial phase a four-point response scale was constructed and reviewed by patients, with the response alternatives ranging from 0 to 3: “No (0), Yes to a certain extent (1), Yes, quite a lot (2) or Yes, a lot (3)” where a higher score implied higher symptom burden.

Concerning the recall time, patients were asked for their most recent experience of arrhythmia symptoms, normal duration of their arrhythmia and length of time of the longest episode. The patients were also asked to estimate the number of occasions they had experienced arrhythmia during the three month period prior to completing the questionnaire. No further recall time was requested in the questionnaire.

### The validation phase

#### Patient population and inclusion criteria

The study inclusion period lasted from May 2009 until late December 2009. Patients eligible for study participation were those referred for RFA treatment with the same diagnoses reported for the initial phase at a University Hospital and patients seeking acute care due to AF at two County Hospitals in Sweden. The RFA patients were consecutively asked about participation and the out-patients were conveniently asked at the time for the acute care visit. Patients were included if they met the inclusion criteria: to be referred for RFA treatment due to different forms of arrhythmias or seeking emergency care due to AF, willing to participate in the study, age 18 years, with sufficient knowledge of the Swedish language and capable to independently fill in the study questionnaires.

### Procedures

All RFA patients received posted study information before the intervention and got study information at the time of admission to hospital, before the scheduled intervention. The acute care AF patients received information at the time of their hospital visit. Together with the questionnaires the patients received instructions on how to complete the questionnaires and were encouraged to ask for help if needed. The questionnaires were completed mostly the day before treatment in RFA patients and for the out-patients at the time of the acute care visit.

### Questionnaires for patient-reported outcomes

The patients completed three questionnaires in the study; The Arrhythmia-Specific questionnaire in Tachycardia and Arrhythmia (ASTA see appendix), Symptom Checklist, Frequency and Severity scale (SCL version 3) and MOS 36-Item Short-Form Health Survey (SF-36 version 1.0).

### Symptom Checklist, Frequency and Severity scale

The SCL is a disease-specific checklist measuring arrhythmia-related symptoms and patients' perception of the frequency and severity of the arrhythmia during the last month. The SCL is mostly used in patients with atrial fibrillation describing arrhythmia-related symptoms, but has also been used in patients with other forms of tachycardia treated with RFA. The SCL consists of 16 items for symptom frequency and scores from 0 to 64 (from never having the symptoms to always having the symptoms) and with 16 items for symptom severity scoring from 0 to 48 (mild to extreme). The higher the frequency score, the greater frequency with which symptoms are experienced and the higher the severity score the greater severity of the symptoms experienced, i.e. lower scores represent better status [15,17,25].

### MOS 36-Item Short-Form Health Survey

The SF-36 was used to assess general health. SF-36 comprises 35 items grouped into eight scales and one question concerning changes in health. The eight scales represent physical functioning (PF), role limitations due to physical health problems (RP), bodily pain (BP), general health (GH), vitality (energy/fatigue (VT)), social functioning (SF), role limitations due to emotional problems (RE) and mental health (psychological distress and psychological well-being (MH)). The eight scales are summarized in two dimensions, physical and mental component summary (PCS/MCS). For each of the eight scales and the two dimensions, scores were coded, summed and transformed to a scale ranging from 0 (worst possible health) to 100 (best possible health). The scoring in SF-36 data was carried out as described by Ware and colleagues [26][29].

### Statistical analysis

Means, standard deviations and frequencies were used to describe the characteristics of the sample and patient symptoms. The ASTA symptom burden questionnaire was psychometrically evaluated regarding data quality, construct validity, concurrent validity and internal consistency reliability.

Data quality was evaluated regarding score distribution and missing data pattern. Frequencies were used to evaluate the distribution of item responses while the Kolmogorov-Smirnov test was used to evaluate if the score in the ASTA symptom scale deviated from a normal distribution. Frequencies were used to describe the missing data pattern and to describe floor and ceiling effects. Floor and ceiling effects were defined if the majority of scores were distributed at either end of the response scale [30].

Homogeneity between the symptoms was evaluated with Spearman´s correlations (r_s_), and internal consistency reliability was evaluated with Cronbach´s alpha coefficient (α) in the ASTA symptom scale [30,31]. An α coefficient ≥0.70 was considered sufficient. In addition, a lower bound confidence interval (95 %) for Cronbach´s alpha was calculated [30,31].

Construct validity was evaluated using item-total correlations adjusted for overlaps and an acceptable level was set to ≥0.30 [30]. Convergent and discriminant validity were evaluated and for this purpose Spearman´s correlation coefficients were used. To support convergent and discriminant validity the ASTA symptom scale was hypothesized to correlate more strongly with SCL’s frequency and severity scales compared to the scales in SF-36.

Concurrent validity was established by Spearman´s correlations between the ASTA symptom scale and the SCL frequency and severity scales.

A p-value of <0.05 was considered significant. Statistical analyses were conducted using SPSS 18 for Windows (SPSS, Inc., Chicago, IL) and Stata 11.1 for Windows (Stata Corporation, College Station, TX).

## Results

### Patient demographics

There were 270 patients included in the validation study, 215 RFA patients and 55 patients seeking emergency care due to AF. The majority of patients were men (66 %) with a mean age of 59 years (range 18–89 years). Beta blockers was the most frequently used anti-arrhythmic medication (59 %), most patients (83 %) were cohabitants and more than half of the patients (63 %) had an educational level of upper secondary school certificate or more, including university degrees (Table 1).

**Table 1:** Characteristics of the participants

		**Radiofrequency catheter ablation - RFA**							**Emergency care**
**Diagnoses and patients**	**TotalN = 270**	**AVNRT n = 40**	**WPWn = 16**	**FATn=8**	**Atrial macro-reentryn = 4**	**AFLn = 16**	**AFn = 126**	**Ventricul ararrhythmian = 5**	**AFn = 55**
**Age mean (SD)**	59.3(12.9)	54.6(13.1)	38.8(15.4)	54.3(17.5)	59.3(10.0)	59.1(15.3)	60.3(9.3)	60.6(13.0)	67.3(10.3)
**GenderF/M(M %)**	93/177(66)	23/17(43)	8/8(50)	5/3(38)	1/3(75)	3/13(81)	33/93(74)	0/5(100)	20/35(64)
**Cohabitant/living alone(cohabit. %)**^**1**^	223/45(83)	35/5(88)	13/3(81)	7/1(88)	3/1(75)	14/2(88)	105/20(83)	4/1(8)	42/12(76)
**Education**^**2**^									
**Elementary school certificate**	26(10)	3(7.5)	1(6)	0	1(25)	0	10(8)	0	11(20)
**Compulsory school certificate**	71(26)	14(35)	2(13)	4(50)	0	2(13)	34(27)	0	15(27)
**Upper secondary school certificate**	96(36)	11(28)	9(56)	3(38)	3(75)	7(44)	44(35)	2(40)	17(31)
**College/University degree**	73(27)	11(28)	4(25)	1(13)	0	7(44)	36(29)	3(60)	11(20)
**Medication**^**3**^									
**Class I(%)**	46(17)	0	1(6)	0	0	0	38(30)	0	7(13)
**Class II(%)**	159(59)	16(40)	6(38)	3(38)	2(50)	10(63)	81(64)	2(40)	39(71)
**Class III(%)**	41(15)	4(10)	0	2(25)	2(50)	1(6)	27(21)	2(40)	3(6)
**Class IV(%)**	36(13)	1(3)	0	0	2(50)	0	24(19)	0	9(16)
**Digitalis(%)**	20(7)	0	1(6)	1(13)	0	1(6)	10(8)	1(20)	6(11)

AVNRT = AV-Nodal reentry tachycardia, WPW = Wolff-Parkinson-White syndrome, FAT = focal atrial tachycardia, atrial macro-reentry, AFL atrial flutter, AF = atrial fibrillation, AF patients with out-patient visits in emergency room. Ventricular arrhythmia includes ventricular tachycardia and VES = Ventricular extra beats. RFA = radiofrequency ablation.

^1^ 2 missing values.

^2^ 4 missing values.

^3^ Here, medication is anti-arrhythmic medication where one patient can have more than one drug.

Class I represents flecainide/propafenon, class II beta blockers, class III amiodarone/sotalol, and class IV calcium channel blockers.

### Data quality

Two symptoms, cold sweat and chest pain, met the criteria for floor effect. No symptom showed ceiling effect. The mean score for the 10 item ASTA symptom scale was 12.2 (SD 5.5) and the scores were normally distributed according to the Kolmogorov-Smirnov test (p = 0.252). Missing data were equally distributed across scale items and were represented by 20 patients having missing answers in all ten items of the scale, one patient with nine missing answers, one had eight missing answers and the other 18 patients had between 1–3 missing answers each.

### Homogeneity and internal consistency reliability

Homogeneity between the 10 items in the ASTA symptom scale was reflected by significant correlations between the items except for three symptoms: worry and breathlessness during activity, infirmity and chest pain and lastly, infirmity and pressure/discomfort in chest.

The rest of the items were significantly correlated with the strongest correlation between the symptoms infirmity and weakness (Table 2). These correlations lead to a revised symptom scale with 9 items, combining weakness/infirmity in one question. The internal consistency was found to be satisfactory high for the revised 9 item scale (α = 0.80) (Table 3) and lower bound confidence interval for Cronbach´s α was satisfactory as well (α = 0.76).

**Table 2:** Correlations between arrhythmia-specific symptoms in ASTA

	**ASTA**	1	2	3	4	5	6	7	8	9	10	11	12	13
1	Breathlessness during activity	1												
2	Breathlessness even at rest	0.60***	1											
3	Dizziness	0.20 **	0.26***	1										
4	Cold sweat	0.21**	0.28***	0.32***	1									
5	Pronounced tiredness	0.42***	0.34***	0.34***	0.29***	1								
6	Chest pain	0.23***	0.27***	0.35***	0.24***	0.20 **	1							
7	Pressure/ discomfort in chest	0.28 ***	0.39 ***	0.28***	0.24***	0.18**	0.57***	1						
8	Worry	0.07ns	0.17**	0.21**	0.16*	0.24***	0.16*	0.21**	1					
9	Weakness	0.40***	0.36***	0.33***	0.33***	0.60***	0.13*	0.16*	0.26***	1				
10	Infirmity	0.41***	0.30***	0.31***	0.31***	0.64***	0.10ns	0.11ns	0.31***	0.68 ***	1			
11	Near syncope	0.24***	0.14*	0.44***	0.20**	0.19**	0.14*	0.09ns	0.08ns	0.22**	0.18 **	1		
12	Syncope	0.10ns	0.02ns	0.10ns	0.09ns	0.14*	0.11ns	0.05ns	0.12ns	0.07ns	0.11 ns	0.47 ***	1	
13	Tiredness afterwards	0.22**	0.23**	0.30***	0.27***	0.62***	0.29***	0.23**	0.16*	0.43***	0.46 ***	0.09ns	0.04ns	1

Statistic correlation analyses were performed using Spearman´s correlation coefficient.

*** p<0.001, **p<0.01 and *p<0.05, ns=not significant.

**Table 3:** Data quality and item-total correlations for ASTA symptom scale

**Items**	**Symptom scale 10 items**^1^			**Symptom scale 9 items**^1^			**Answer alternatives**^2^			
**Symptoms in connection with arrhythmia**	**Item-total correlation**	**Cronbach´s α if item deleted**	**MeanSD**^1^	**Item-total cor*r*elation**	**Cronbach´s α if item deleted**	**MeanSD**^1^	**“No”n(%)**	**“Yes, to a certain extent” n(%)**	**“Yes, quite a lot” n(%)**	**“Yes, a lot” n(%)**
Breathlessness during activity	0.496	0.808	1.79(0.98)	0.479	0.777	1.79(0.98)	24(9)	75(28)	73(27)	70(26)
Breathlessness even at rest	0.569	0.801	0.77(0.81)	0.588	0.762	0.77(0.81)	105(39)	107(40)	22(8)	11(4)
Dizziness	0.468	0.810	0.86(0.79)	0.465	0.778	0.86(0.79)	87(32)	116(43)	33(12)	10(4)
Cold sweat	0.455	0.813	0.97(0.98)	0.450	0.781	0.97(0.98)	100(37)	76(28)	48(18)	22(8)
Pronounced tiredness	0.604	0.795	1.74(0.98)	0.544	0.767	1.74(0.98)	26(10)	75(28)	79(29)	65(24)
Chest pain	0.431	0.814	0.56(0.74)	0.465	0.778	0.56(0.74)	134(50)	91(34)	14(5)	7(3)
Pressure/discomfort in chest	0.428	0.814	1.01(0.88)	0.461	0.778	1.01(0.88)	74(27)	116(43)	37(14)	18(7)
Worry	0.367	0.820	1.29(0.86)	0.348	0.793	1.29(0.86)	39(14)	125(46)	57(21)	27(10)
Weakness	0.650	0.792	1.54(0.86)	0.585	0.762	1.54(0.86)	21(8)	115(43)	73(27)	38(14)
Infirmity	0.609	0.795	1.67(0.92)	-	-	-	21(8)	93(34)	80(30)	53(20)
		**Cronbach´s α0.822**	**12.18(5.48)**		**Cronbach´s α0.795**	**10.51(4.88)**				

^1^ n = 230.

^2^ n = 242–247, the number of missing answers varied between 23–28 for the different items in the 10 item scale.

The association between symptoms outside the ASTA symptom scale and symptoms in the scale demonstrated one strong correlation and this was between the symptoms exploring tiredness; tiredness afterwards and pronounced tiredness during arrhythmia (r_s_ > 0.6) (Table 2).

### Construct validity

The 9 items in the revised symptom scale reached the expected level of item-total correlations, ranging from 0.35 (worry) to 0.59 (breathlessness even at rest), indicating the items measuring the same concept (Table 3).

Convergent and discriminant validity was demonstrated for the ASTA symptom scale where the correlations were more strong with the SCL frequency and severity scales (r_s_ > 0.60) than with the scales in SF-36, supporting convergent validity. The ASTA symptom scale correlated weaker but significantly (r_s_ < 0.60) with the SF-36 eight scales and physical and mental component summary, supporting discriminant validity (Table 4). Strong correlations were found between the SCL scales and the scales in SF-36. The lowest correlation was seen for role-emotional and the SCL severity scale, r_s =_0.43, and strongest correlation, r_s =_0.70 for vitality and the SCL frequency scale.

**Table 4:** Correlations between ASTA symptom scale, SCL and SF-36

		1	2	3
1	**ASTA**^1^Symptomscale	1		
2	**SCL** Frequency	0.62***	1	
3	Severity	0.61***	0.94***	1
4	**SF-36** PF	−0.32***	−0.57***	−0.56***
5	RP	−0.25***	−0.50***	−0.49***
6	BP	−0.39***	−0.48***	−0.55***
7	GH	−0.29***	−0.47***	−0.45***
8	VT	−0.44***	−0.70***	−0.63***
9	SF	−0.31***	−0.51***	−0.49***
10	RE	−0.32***	−0.46***	−0.43***
11	MH	−0.33***	−0.59***	−0.53***
12	PCS	−0.29***	−0.54***	−0.55***
13	MCS	−0.36***	−0.54***	−0.50***

^1^ The revised ASTA symptom scale with 9 items. Statistic correlation analyses were performed using Spearman´s correlation coefficient. *** p < 0.00.

SCL symptom frequency and severity scale. The eight scales in the SF-36 includes PF = Physical Functioning, RP = Role-Physical, BP = Bodily Pain, GH = General Health, VT = Vitality, SF = Social Functioning, RE = Role-Emotional, MH = Mental Health and PCS = Physical Component Summary, MCS = Mental Component Summary.

### Concurrent validity

Concurrent validity was demonstrated with strong correlations (r_s_ > 0.60) between the ASTA symptom scale and both scales in the SCL frequency and severity scales (Table 4).

### Patients’ symptoms

The most commonly reported symptoms by the patients were weakness, infirmity and breathlessness during activity and pronounced tiredness, and these symptoms were also the most burdensome for the patients (mean ≥1.5, Table 3). Chest pain, breathlessness even at rest, dizziness and cold sweat were the symptoms more seldom reported and less burdensome for the patients (mean ≤1.0, Table 3). The patients were asked about experiences of palpitation during arrhythmia episodes and many of the patients felt rapid heart rhythm (71 %), most experienced the arrhythmia as being irregular (61 %) . Common during arrhythmia episodes was a feeling of harder heart beats (43 %) and missing beats (45 %). The patients were also asked to estimate the duration of their arrhythmia and most common were episodes shorter than seven hours (40 %), but some (12 %) had duration of more than 48 hours. The frequency of arrhythmia during the three month period before treatment varied between not having any arrhythmia at all (8 %) to having episodes more often than 15 occasions or experiences of arrhythmia daily (36 %). Many patients were tired afterwards (74 %) and some of the patients had experienced the more disabling symptoms near syncope and syncope (32 % and 11 % respectively) in connection with the arrhythmia.

Thirty percent of the patients answered that the arrhythmia appeared on special occasions. The occasions mentioned were: At stress, during nights, at effort/physical activities, rapid movements, anxiety, when angry, when forgotten the medication. Seventeen percent answered “yes” to food or drinks as having an influence on arrhythmia appearance. Most commonly contributing to the onset of the arrhythmia was alcohol, then coffee, but fat or spicy foods and eating too much was also mentioned. More than half of the patients, 52 %, were not able to interrupt the arrhythmia by self-management after the onset.

## Discussion

The new ASTA symptom questionnaire showed generally good reliability and validity properties.Strengths with the study were the amount of patients and the repeated expert panel evaluations. This increases the possibility to have covered the most relevant and commonly experienced symptoms and to have achieved a scale with good sensitivity [32]. Although the ASTA symptom scale includes rather few symptoms the scale is likely to have sufficient coverage and relevance in different forms of arrhythmias. Too few items increases the risk for loss of information, on the contrary too many can lead to a risk for measuring the same construct but in a slightly different way and make it burdensome to complete [33].

Data skewness was not a problem even though two symptoms showed floor effect and the symptoms showed good item-total correlation to the symptom scale [30]. The concerns about floor and ceiling effects is regarding the ability to discriminate between healthier patients and patients with more severe conditions and also difficulties with sensitivity and responsiveness to changes [32]. The scale had an overall good distribution of the scores, implying the symptoms being able to discriminate between patients with a high respectively low symptom burden.

The missing data pattern showed an equal distribution of missing values. Except those with missing values for all the symptoms in the symptom scale, the overall missing data was low [32]. Some of the patients did not experience distinct arrhythmia-related symptoms and were therefore not motivated to complete the questionnaire. This was the case in an earlier validation study where patients were asked about limitations in working capability and where rather many had difficulties to respond because they were retired [16].

The correlations between the items in the ASTA symptom scale showed generally satisfactory homogeneity. All items correlated to the total scale and the internal consistency was sufficiently as well as the lower bound confidence interval for Cronbach´s α. A reasonable explanation for the strong correlation between infirmity and weakness was that these two probably described almost the same concept. In the literature there are items described as causal or effect indicators and there is an ongoing debate concerning different types of scales, clinimetric and psychometric scales [32,34,35]. Causal indicators can be symptoms that affect QoL and scales can be described as symptom scales including a variety of items, not to be expected to satisfy the same demands as for psychometric scales [32,34]. However, many items may be both causal and effect in nature and statements concerning distinctions between different scales have been criticized to be just confusing and limiting [35,36]. We still emphasize that homogeneity matters as the ASTA symptom scale aims to measure the concept symptom burden.

Construct validity was supported by the sufficient item-total correlations where all except one reached the more stringent criteria suggested for final testing of scales (>0.4) [32]. Convergent and discriminant validity are important aspects of construct validity [32,35]. We expected a stronger correlation between the ASTA symptom scale and the arrhythmia specific SCL and significant but weaker correlations with the generic SF-36, the latter because both explore physical and mentally-related domains [16,37][39].

At the time of the validation of ASTA, SCL was the most well-known and commonly used questionnaire in arrhythmia patients and therefore also chosen to represent the “golden standard” for criterion validity.

The hypothesis was confirmed but we had however expected to find an even stronger correlation between ASTA and the two scales within SCL. Further we found that SCL had a strong correlation with one subscale in SF-36. This can be explained by that SCL includes more general symptoms, for example headache, poor appetite, nausea and also consequences on patients’ daily life such as problems to concentrate and difficulty to sleep whereas the ASTA symptom scale was developed for assessing arrhythmia-specific symptoms [15,17,25]. Overall, rather strong correlations were found between SF-36 and the SCL scales supporting the more general nature of the symptoms in SCL. However, the most pronounced correlation was seen between the two scales within SCL, indicating the two separate scales measuring almost the same concept.

More than one third of the patients in the validation study had been near syncope and more than every 10th patient had experienced syncope in connection with arrhythmia, and it would have been valuable to further explore how often the patients had suffered from these disabling symptoms, but this information is unfortunately not available. Presence of severe symptoms in arrhythmia patients has been described in earlier studies as well as the consequences due to a feeling of insecurity, leading to self-imposed daily life restrictions [7,40].

The patient groups aimed to be covered by the ASTA questionnaire were included in the validation process. However, in some groups there were few patients and therefore, it was not relevant to examine know-groups validity.

During a development phase it is natural and important to make relevant changes but unfortunately this reduced the number of patients available for inclusion in the validation study.

Even if the symptom items in ASTA are relevant and not too lengthy and probably not too burdensome for the patients, there will be a need for a system to handle missing data. We did not want to make any imputation because this is a validation study but we have rewritten the patients user instructions to more clearly state how to complete the questionnaire.

The initial tests with the combination of worry/anxiety (within the initial phase not reported here) showed higher internal consistency and item-total correlation for the combination and for the revised ASTA symptom scale these symptoms will be reworded to asking for worry/anxiety. In the revised ASTA we further ask for tiredness in connection with arrhythmia and have erased the wording pronounced because the answer alternatives already give the grade of severity, i.e. if it is pronounced or not. Tiredness after having arrhythmia was commonly experienced but excluded in the revised version, where the remaining symptoms are all asked for in connection with the arrhythmia episodes.

Treatment options have been extensively developed during the last decades with upgraded international guidelines and new anti-arrhythmic agents available [1]–[3,41]. It is important to assess frequently experienced symptoms and also the most distressing symptoms for the patients.

Interventions can be curative or preliminarily expected to affect the symptom burden with medical treatment, sometimes life-long, to achieve freedom and relief from symptoms and for improving the patient’s daily life [4]. Instead of focusing solely on ECG-measurement outcomes the primary goal must be the patient’s well-being, the relief of symptoms or symptom control. What are we treating, the patient or the ECG? [12].

Measurement of subjective well-being is essential in order to explore the severity of a disease and to evaluate therapeutic efficacy both in clinical and research settings [9,10].

The ASTA symptom questionnaire was developed together with the experts themselves e.g. the arrhythmia patients and health care professionals with long time experiences of working with arrhythmia patients.

For the usefulness of ASTA as a working tool in clinical practice and for research settings we have striven for user friendly instructions. The instructions clearly guide the patients how to respond if they feel symptomatic or not. We have performed several of the recommended psychometric analyses to see to the reliability and validity of the new questionnaire, but it needs to be further tested to confirm test-retest reliability and responsiveness to changes.

Even if there are some newly developed questionnaires [13,16,21,37] since the start of the ASTA project none of them evaluates symptom burden in most of the existing forms of arrhythmia.

The advantages with the new ASTA symptom burden questionnaire are that it is suitable for most forms of arrhythmias, assesses symptom burden separately and includes not solely different symptoms, but also the duration and the frequency of the episodes. In the ASTA questionnaire both commonly experienced symptoms as well as less frequent but severe and distressful symptoms are thoroughly evaluated.

So far only the Swedish version has been validated but hopefully, in cooperation with the corresponding author, the questionnaire will be properly forward-backward translated and validated in several other languages.

## Conclusions

The ASTA symptom scale was found to have good psychometric properties in patients with different forms of arrhythmias. Arrhythmia patients suffer from both frequent and disabling symptoms and PROs are important to assess, when evaluating these patients’ life situation. The newly validated ASTA questionnaire can be an important contribution to PROs in arrhythmia patients.

## Authors' contributions

UW: Study design, instrument development, main responsible for manuscript writing, data analysis.AS: Study design, instrument development, manuscript preparation, supervision. KÅ: Study design, data analysis, manuscript preparation, supervision. All auhtors read and approved the final manuscript.

## Competing interests

The authors declare that they have no competing interests.
